# Comparing Intelligence Quotient (IQ) Among 3 to 7-Year-Old Strabismic and Nonstrabismic Children in an Iranian Population

**DOI:** 10.5539/gjhs.v8n3p26

**Published:** 2015-06-25

**Authors:** Mahboubeh Ghaderpanah, Feraidoon Farrahi, Gholamreza Khataminia, Ahmad Jahanbakhshi, Leila Rezaei, Ashraf Tashakori, Mohammad Mahboubi

**Affiliations:** 1 Department of Ophthalmology, Ophthalmology Research Center, Jundishapur University of Medical Sciences, Ahvaz, Iran; 2 Department of Ophthalmology, Kermanshah University of Medical Science, Kermanshah, Iran; 3 Psychiatric Ward of Golestan Hospital, Jundishapur University of Medical Sciences, Ahvaz, Iran; 4 Abadan School of Medical Sciences, Abadan, Iran

**Keywords:** intelligence quotient, strabismus, deviation

## Abstract

This study was designed to compare the Intelligence Quotient (IQ) among 3 to 7-year-old strabismic and nonstrabismic children in an Iranian population.

In this cross-sectional study, 108 preschool children with equal numbers of strabismic/non-strabismic disorder (age 3–7 years) were randomly selected from exceptional strabismus clinics of Ahvaz and were evaluated with the preschool and primary scale of intelligence versions of Wechsler (WPPSI).

In the current study, 108 children were evaluated. In strabismic patients the mean performance, verbal and total IQ were 89.46±19.79, 89.57±21.57 and 91.54±22.08 respectively. These mean scores in normal children were 91.89±47.53, 87.56±15.6 and 89.96±17.62consecuently. The results showed that these three different IQ subscales were not significantly different among 3 to 7 years old strabismic and nonstrabismic children ((P>0.05 for all comparisons). There was no significant difference in IQ between two sexes (P>0.05) while Persian tribe children had greater IQ score compared to other tribes (P<0.05). Also, higher paternal educational status of children related to higher IQ score. IQ score was better in combined deviations and was higher in exotropes than esotropes; however, these differences were not statistically significant (P>0.05).

In this evaluation, we did not found a significant negative interference of strabismus on IQ score of preschool children. It can be concluded that paternal educational level and tribe have a significant effect on intelligent quotient, while this is not the case on sex and ocular deviation.

## 1. Introduction

Strabismus (ocular misalignment) is one of the ophthalmological problems that can affect the quality of life of individuals. Patient with strabismus fail to achieve proper binocular vision because they are unable to simultaneously direct each eye to the same point in space. In addition, the appearance of misaligned eyes might result in social prejudice by associating strabismus with personality defects and below average intelligence (Olitsky, Sudesh, Graziano, Hamblen, Brooks, & Shaha, 1999). Prejudice relating to strabismus can extend beyond social relationships. Adult with strabismus are likely to develop mannerisms to camouflage their dysfunction and avoid eye contact during social interactions (Nelson, Gunton, Lasker, Nelson, & Drohan, 2008; Durnian, Owen, Baddon, Noonan, & Marsh, 2010). They also perceive that strabismus has a negative impact on secure employment and opportunities for career advancement (Durnian et al., 2010).

The main purpose of strabismus treatment is the alignment of the visual axes in order to achieve single binocular vision with good image fusion. Other advantages of strabismus correction include the improvement of any abnormal head posture, expansion of the visual field, restoration of stereoscopic acuity, centralization of the visual field, elimination of diplopia, improvement in ocular motility, improvement in psychomotor development, and restoration of normal appearance (Olitsky et al., 1999; Nelson et al., 2008). Most aspects of development are dependent or guided by visual system. So, identifying the effective factors on intelligence and early treatment of them in children can prevent learning disorders and secondary problems in children.

Within the past few years, psychological issues of children have received a lot of attention. Egeland et al obtained a great correlation between childhood behavioral disorders and behavioral disorders of adulthood (Egeland, Pianta, & Ogawa, 2004). Different plans have been designed in order to identify risk factors which are specific to each disorder and selecting population which is prone to danger in order to conduct interventions. However, such plans are expensive and involve specialized screening (Do Zois & Dobson, 2006). Behavioral disorders in children bring many problems for society because children behavior influences on their family, teachers and everyone who has close relationship with children. The final losers of such effects are children themselves. On the other hand, behaviors of such children have negative influence on their education process and insignificant advancement in academic area is the immediate resultant of such behaviors (Egeland et al., 2004; Do Zois & Dobson, 2006).

Previous studies on the intelligence and psychosocial negative effects of strabismus in adults were published (6). However, to our knowledge only one no controlled study have investigated the Intelligence Quotient (IQ) (without attention to age differences) among strabismic patients in Iran. The aims of this study were to compare the IQ properties in 3 to 7-year-old preschool children with or without strabismus in the another portion of Iranian population.

## 2. Methods

### 2.1 Study Design

Cross sectional, case control, prospective study.

### 2.2 Ethical Consideration

All study procedures were conducted in accordance with the Declaration of Helsinki. A participant information sheet and verbal explanation were given. This is an anonymous questionnaire, so no personally identifiable information of patients and parents was recorded. The consent to participate in this study was assumed upon the completion of this questionnaire (i.e., completing the questionnaires implies giving consent to participate). The institutional ethics committee approved the study, and all patients provided written informed consent.

### 2.3 Participants

A consecutive sample of 54 child patients with strabismus was visited at strabismus clinic of Emam khomeini eye hospital (Ahvaz, Khuzestan, Iran). They were attending the hospital in connection with their strabismus, possibly with a view to having strabismus surgery. At the time of the study, the patients were interviewed in the strabismus outpatient department. After examination by ophthalmologist; patients who met surgical indications were added to a waiting list for a strabismus surgery.

In this study, inclusion criteria for strabismus patients were: aged between 3-7 years; no history of any eye related surgery before participation or any diagnosed emotional or anxiety disorders; no other facial or ocular abnormalities or metabolic or neurologic diseases; good cooperation of children for IQ test and the angle of deviation was no less than 15 prism diopters (PD).

A control group of 54 children without any visual defect were recruited. To ensure similar baseline conditions, subjects were all from the family of strabismic patients. Visually normal child were companions or family members of the patients in the eye clinic.

### 2.4 Questionnaires

The preschool and primary scale of intelligence versions of Wechsler (WPPSI) have been developed following standard processes of translation to Persian (Razavieh & Shahim, 2008). Questionnaires include items that are designed to be relevant to a particular group of ages, and are therefore more sensitive to small but clinically significant changes in IQ in either group.

Khuzestanian population include three peculiar peoples: Persian, Arab, and Bakhtyari tribe, so, this test performed by three different psychologists (Persian speaking, Arabic speaking, Bakhtyari speaking). Specifically, either psychologists who were predominate to first language of patients performed their IQ test. All of the test portions were discussed by an expert pediatric psychologist.

This test included two portions: performance and verbal intelligence:

1). The performance subscale contained 6 items: animal house (with 1-3 time repetition), picture completion (23 pictures), geometric design, block design, picture arrangement, object assembly coding.

2). The verbal subscale contained 7 different items: information, vocabulary (22 words), arithmetic, similarities (16 items), comprehension (15 items), sentences and digit span.

Answers for each item was recorded in a rating scale. From these crude scales, a verbal intelligence score, performance intelligence score and total intelligence score was calculated and recorded in special form.

The average or normal, range of IQ is 90 to110; IQ scores of at least 120 are considered superior. Mental retardation is defined as an IQ below 70, which corresponds to the lowest 2.2 percent of the population (B. J. Sadock & V. A. Sadock, 2007).

### 2.5 Data Collection

All data were collected among strabismus patients prior to any strabismus-related surgery. Researchers emphasized that participation was entirely voluntary and the choice to participate or not had no impact on their surgery or treatment.

For the purpose of standardization, the questionnaires were bound in a fixed order.

A subgroup of 54 nonstrabismic children was randomly selected from the strabismic patient's family who matched in sex and age. Demographic information such as age, sex, and special first language, educational level of the parents, their domicile, the type of deviation, angle of deviation in prism diopter (PD) and refractive error (hyperopic and myopic) was recorded.

### 2.6 Statistical Analysis

Statistical analysis was performed using SPSS software (version 18, SPSS Inc). Data is expressed as mean ± SD. A p -value of less than 0.05 is considered to be statistically significant. We compared the mean score in strabismic patients, with that in visually normal children using t-test, Pearson correlation coefficient and ANOVA test.

## 3. Results

### 3.1 Demographic and Baseline Characteristics

A total of 108 questionnaires were fully or correctly completed. Thus 108 valid demographic and Wechsler questionnaires were available for statistical analysis.

In strabismic group: 25 patients (46.3%) were female and 29 patients (53.7%) were male.

In nonstrabismic or normal group: 31 children (57.4%) were female and 23 children (42.6%) were male.

The mean age of strabismic and normal children were 5.1 ± 1.12 and 5.1±1.09 years, respectively; range, 3.9-6.6 years. No statistically significant differences were found between the study groups (strabismic patients, visually normal children) in terms of age, gender, and socioeconomic status (Table 1).

**Table 1:** The frequency and percentage of children in both groups based on sex (n=108)

Sex	Strabismic group		Normal group	
	
	Frequency	Percentage%	Frequency	Percentage%
Female	25	46.3	31	57.4
Male	29	53.7	23	42.6
Total	54	100	54	100

In strabismic group 46 children (85%) were hyperopic and 8 children (15%) were myopic. The mean refractive error in hyperopic eyes were+3.46±2.65 dpt (ranging from+1.75 to +5.25 dpt). The mean refractive error in myopic eyes were-3.68±2.01 dpt (ranging from-1.00 to –8.25 dpt).

From 54 families that their strabismic and nonstrabismic children participated in this study only 15 mothers (27.7%) and 16 fathers (29.7%) of them hold a university certificate and above, while about 70% reported an education level below high school diploma. All children of present study had both father and mother and financial and emotional support of them.

**Table 2:** Frequency and percentage of educational level of parents

Educational level	Maternal educational level		Paternal educational level	
	
	Frequency	Percentage%	Frequency	Percentage%
Illiterate	9	16.7	5	9.2
Elementary	9	16.7	10	18.5
High school diploma	21	38.9	23	42.6
Associated degree	5	9.2	5	9.2
Graduate and more	10	18.5	11	20.5
Total	54	100	54	100

In this study twenty six of families (48.1%) were Bakhtiyari tribe, 15 families (27.8) were Arabic tribe and 13 other families (24.1%) were Persian tribe.

### 3.2 Relationship Between Intelligence Quotient (IQ) Scores of Study Groups Compared to Normal Population

Intelligence quotient (IQ) scores of two study groups compared to normal Iranian population are showed in Tables 3 and 4.

**Table 3:** Iranian population standard of Wechsler intelligence Quotient (IQ) (Razavieh & Shahim, 2008; B. J. Sadock & V. A. Sadock, 2007)

Level of IQ	Intelligence Quotient	Theoretical percentage	Real percentage
Very superior	>130	2.2	2.4
Superior	120-129	6.7	8.1
Bright	110-119	16.1	17.1
Normal or Average	90-109	50	47.1
Dull or Subnormal	80-89	16.1	15.3
Borderline	70-79	6.7	7.5
Mental retard	<70	2.2	2.2
Total	-	100	100

**Table 4:** Intelligence quotient (IQ) scores in the study population compared to a normal Iranian population

IQ level	Expected percentage in Iranian population (%)	Observed frequency and percentage in patients (%)	Difference (=Observed percentage ”Expected percentage).
Superior elite	2.2	2 (3.7)	+1.5
Elite	6.7	7 (13)	+6.3
Bright	16.1	7 (13)	-3.1
Average or Normal	50	8 (14.8)	-35.2
Dull or Subnormal	16.1	12 (22.2)	+6.1
Borderline mental retard	6.7	10 (18.5)	+11.8
Mental retard	2.2	8 (14.8)	+12.6

According to histogram 1, 2 total IQ score distribuation in strabismic children do not have significant different from normal distribution. (Histogram 1, 2)

**Histogram 1:**
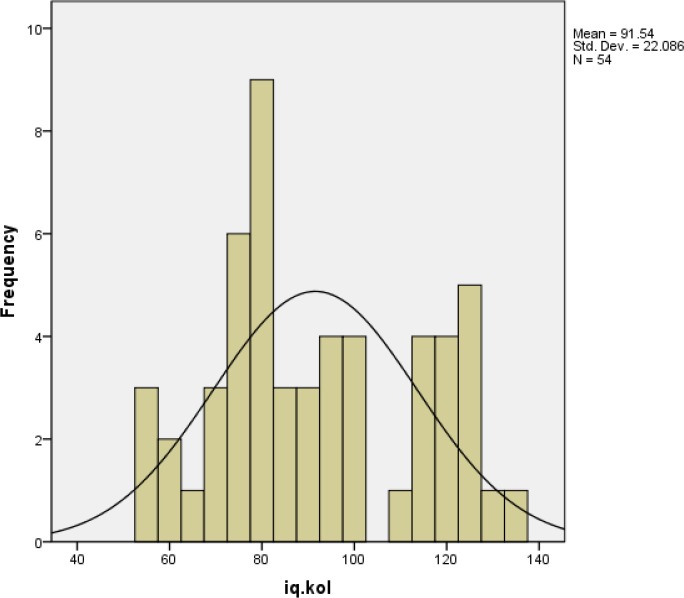
Distribution of total intelligence quotient (IQ) scores in the strabismic group compared to the normal population

**Histogram 2:**
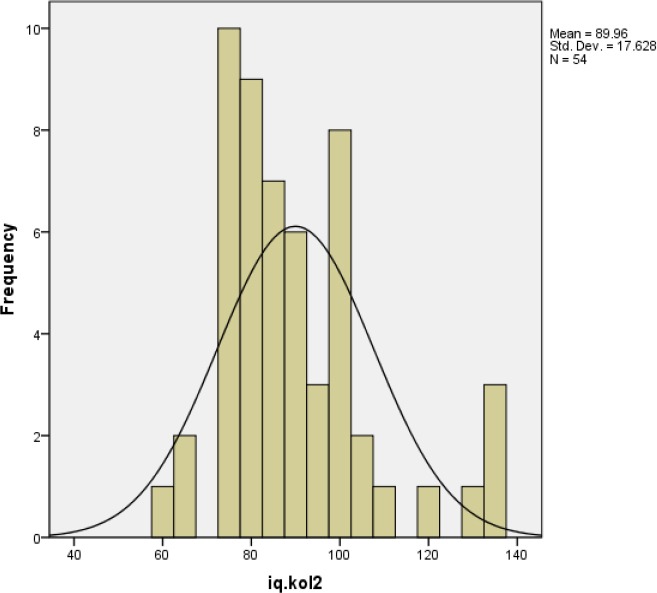
Distribution of total intelligence quotient (IQ) scores in the nonstrabismic (normal) group compared to the normal population

In strabismic patients the mean verbal IQ were 89.46±19.79 and the mean performance IQ were 89.57±21.57. There was no significant difference between two IQ scores in strabismic patients (P-value =0.95).

In normal children the mean performance IQ and verbal IQ were 91.89±47.53 and 87.56±15.6 respectively that did not have significant difference (P-value=0.49).

We did not found statistical different in IQ scores of both strabismic and normal groups (all p-value>0.05) (Table 5).

**Table 5:** Mean and SD of intelligence quotient (IQ) in two study groups based on three different IQ subscales

Groups	Verbal IQ	Performance IQ	Total IQ
Strabismic	89.46±19.79	89.57±21.57	91.54±22.08
Normal	87.56±15.6	91.89±47.53	89.96±17.62
t-test (P-value)	0.55 (0.5)	0.32 (0.74)	0.68 (0.49)

### 3.3 Relationship between Intelligence Quotient (IQ) and Sex

In 54 strabismic children (25 female and 29 male) the mean IQ scores of female were higher than male, however this difference were not statistical significant (P-value >0.05) (Table 6).

**Table 6:** Mean and SD of intelligence quotient (IQ) in different IQ category based on different sex

Groups	Verbal IQ	Performance IQ	Total IQ
Female	90.19±16.69	96.61±60.33	91.77±15.88
Male	84.00±13.55	85.52±20.11	87.52±19.84
t-test (P value)	1.47(0.15)	0.84 (0.4)	0.87 (0.38)

### 3.4 Relationship between Intelligence Quotient (IQ) and Tribe

In 54 strabismic patients (26 Bakhtiyari, 15 Arab, 13 Persian) the verbal IQ in Bakhtiyari, Arab, Persian were 90.69±16.93, 75.73±21.04 and 102.85±13.56 respectively and this difference were statistically significant (P=0.001). Other performance and total IQ of these three tribes were different statistically (P value=<0.05) (Table 7).

**Table 7:** Mean and SD of intelligence quotient (IQ) in different IQ category based on different tribes

Tribes	Frequency (Percentage %)	Verbal IQ	Performance IQ	Total IQ
Bakhtiyari	26(48.1)	90.69±16.93	84.88±16.15	92.50±19.76
Arab	15(27.8)	75.73±21.04	82.40±21.09	77.80±19.14
Persian	13(24.1)	102.85±13.56	107.23±23.32	105.46±21.55
ANOVA(P-value)	-	8.5 (0.001)	7.13 (0.002)	6.69 (0.003)

### 3.5 Relationship Between Intelligence Quotient (IQ) and Paternal Educational Level and Child Age

Furthermore, Pearson momentum correlation coefficient investigated to study the relation between paternal educational level and IQ subscales. The correlation between paternal and maternal educational level and three different subscale of IQ was significant. Moreover, there was significant correlation between children age and IQ score (P= 0.01 for all comparisons) (Table 8).

**Table 8:** Pearson correlation coefficient to show the relation between paternal educational level and child age and three different intelligence quotient (IQ) subscales

Variable	Verbal IQ	Performance IQ	Total IQ
Maternal education	0.58 (0.01)	0.49 (0.01)	0.63 (0.01)
Paternal education	0.53 (0.01)	0.37 (0.01)	0.53 (0.01)
Children age	0.65 (0.01)	0.45 (0.01)	0.58 (0.01)

### 3.6 Relationship between Intelligence Quotient (IQ) and Ocular Deviation Types

With regard to the type of strabismus, 94% of patients had horisontal deviation (74% esotropia, 20% exotropia) and 6% combined deviation (vertival and horizontal). The patients with exotropia recorded better IQ scores than patients with esotropia in the performance IQ, the verbal subscale and in the total subscale of IQ. Although patients with combined deviation have better performance and total IQ, but these difference were not statistically significant (p-value >0.05 for all comparisons) (Table 9)

**Table 9:** Mean and SD of different intelligence quotient (IQ) category based on different ocular deviation types

Type of deviation	Frequency (Percentage%)	Verbal IQ	Performance IQ	Total IQ
Esotropia	40(74)	87.43±20.44	85.5±19.68	88.63±21.87
Exotropia	11(20)	95.50±16.13	99.40±22.37	98.70±18.30
Combined	3(6)	94.75±22.23	105.75±28.24	102.75±30.71
ANOVA(P-value)	-	0.81 (0.44)	3.1 (0.06)	1.41 (0.25)

The patients with constant deviation recorded better IQ scores than patients with alternate in all of IQ subscales, but these difference were not statistically significant(p-value >0.05 for all comparisons) (Table 10).

**Table 10:** Mean and SD of different intelligence quotient (IQ) score in constant and alternate strabismus

Deviation	Frequency (Percentage%)	Verbal IQ	Performance IQ	Total IQ
Constant	12(22)	96.25±17.43	92.92±24.05	96.33±22.49
Alternate	42(78)	87.52±20.18	88.62±21.03	90.17±22.04
t-test (P-value)	-	1.35 (0.18)	0.60 (0.54)	0.85 (0.39)

### 3.6 Relationship between Intelligence Quotient (IQ) and Amblyopia

Among of 54 strabismic patients, 30 patients (55.5%) had amblyopia, of whom 10 had unilateral (18.5%) and 20 had bilateral (37%) amblyopia. Mean IQ score in relation to amblyopia is presented in Table 12. Even though amblyopic patients had higher IQ levels than non-amblyopic counterparts, there was no significant difference in this regard (P>0.05). (Table 11)

**Table 11:** Frequency of different types of amblyopia and relation between amblyopia and intelligence quotient (IQ) scores

Amblyopia	Frequency (Percentage%)	Verbal IQ	Performance IQ	Total IQ
Unilateral amblyopia	10(18.5)	92.3±19.58	89.00±22.14	92.5±23.06
Bilateral amblyopia	20(37)	89.55±16.56	94.1±20.49	90.4±21.17
No amblyopia	24(44.5)	88.21±22.82	86.04±22.43	92.08±23.31
ANOVA, P-value	-	0.14 (0.86)	0.75 (0.47)	0.04 (0.95)

## 4. Discussion

Intelligence can be defined as the ability to assimilate factual knowledge, to recall either recent or remote events, to reason logically, to manipulate concepts, to translate the abstract to the literal and the literal to abstract, to analyze and synthesize forms, and to deal meaningfully and accurately with problems and priorities deemed important in a particular setting. The most useful intelligence test must measure a variety of skills and abilities including verbal and performance, early learned and recently learned, timed and none timed (Razavieh & Shahim, 2008). Finally, the comparison of the findings of many studies for investigation of reliability and validity of intelligence tests and cognitive abilities indicates that the Wechsler is the best standardized and most widely used intelligence test in our clinical practice today (B. J. Sadock & V. A. Sadock, 2007; Ebrahim & Kamkari, n.d.; Sadeghi, Rabiee, & Abedi, 2011).

The main purpose of this study was to compare intelligence quotient (IQ) of strabismic children and healthy non strabismic preschool children in a Khuzestanian population.

Akinci A et al found that the children with intellectual disability had significantly more nystagmus, strabismus, astigmatism, and hypermetropia than controls. Children with syndromic intellectual disability had significantly more nystagmus, strabismus, astigmatism, and hypermetropia than subjects with nonsyndromic intellectual disability. Increasing severity of intellectual disability was related to higher prevalence of nystagmus, strabismus, astigmatism, hypermetropia, and anisometropia (Akinci, Oner, Bozkurt, Guven, Degerliyurt, & Munir, 2008).

A pleiotropic relationship between intelligence and myopia has been shown to exist. Large eyes (as measured by axial length) have been shown to lead to myopia, and large brains have been shown to be more intelligent. Many authors showed that the myopia/intelligence relationship could arise because a single genetically controlled mechanism affects both brain size and eye size (Tay, Au Eong, Ng, & Lim, 1992; Miller, 1992; Teasdale, Fuchs, & Goldschmidt, 1988).

Sayyadi S et al compared visual perceptual skills among 8 to 10-year-old strabismic and nonstrabismic cerebral palsy (CP) children. They found that non-strabismic CP children had greater visual perceptual quotient compared to strabismic one. They concluded that age and strabismus have a significant effect on visual perceptual quotient (Sayyadi, Lajevardi, Aliabadi, Keihani, & Abbasi, 2011).

Rosner M et al conducted a nationwide study of the relationship among refractive error, intelligence scores, and years of schooling in 157,748 males aged 17 to 19 years, they found a strong association of myopia with both intelligence and years of school attendance (Rosner & Belkin, 1987).

Reports of the psychosocial negative effects of strabismus in adults were published in litrature; the patients said that every aspect of their lives was affected by strabismus, such as self-esteem, employment prospects, interpersonal relationships, education, and playing sports. A study determined that 41.3% patients with strabismus developed mental health problems compared with 30.7% from the control group (Mohney, B. G., McKenzie, Capo, Nusz, Mrazek, & Diehl, 2008; Chua & Mitchell, 2004).

We found only one article in literature that determined IQ score in strabismic patients objectively. Bagheri et al carried out a study in Tehran, Iran on patients with age range of 4-63 years, it was reported that the total IQ score was significantly lower in comparison to the normal population. (6) However, they did not evaluate IQ score of children and adult separately and we did not observed a significant impact of strabismus on the IQ score of the interviewed children.

The results of our study indicate that no significant differences in any of IQ score are evident between males and females, however males in the Chinese sample obtained significantly higher IQ scores than females. These observations were not present in the Japan and United States samples (Liu & Lynn, 2011).

Analysis of the effect of parents’ education level on the three different subscale of IQ showed that the correlation between paternal and maternal educational level and IQ were significant. Like Bagheri et al study it was observed that about70% of parents have the education level below high school diploma, but their results were completely different with those obtained in the current study. They showed that a positive correlation between educational level of the patients and their IQ with no correlation between the level of parents’ education and patients’ IQ score (Bagheri et al., 2013). With regard to the difference in the age range of the two studies participants, his results were different with those obtained in our study and suggested that the effects may be due to an effect of genetic factors and parents financial support on memory of children (B. J. Sadock & V. A. Sadock, 2007). Moreover, there was significant correlation between children age and IQ score in the present study (Table 8).

There were statistically significant differences between tribes and IQ scores in our study groups. In this study, it was found that all performance, verbal and total IQ were significantly higher in Persian tribe, we know the predominant language in kindergarten, cinema and other public spaces of Ahvaz is Persian. So Arabic or Bakhtyari tribe preschool children may showed some difficulty in the test answering.

We found that the effect of the tribe and paternal educational level was greater than the ocular misalignment.

Previous studies reported that strabismic patients with vertical deviations were more intelligent than patients with horizontal deviations; this may be due to compensatory mechanisms such as abnormal head posture (Bagheri et al., 2013). Our results support this concept, partly. In this present study, it has been shown that a minimal, non significant, effect of combined vertical and horizontal ocular deviation in IQ score compared with horizontal deviation alone were found among strabismic children.

Consistent with previous studies, our results indicated that patients with esotropia would more often show lower IQ score compared to exotropic patients. Olitsky SE and colleagues for example, found a negative association between psychosocial characteristics and strabismus and children with esotropia were rated more negatively than those with exotropia on most of these characteristics (Olitsky, Sudesh, Graziano, Hamblen, Brooks, & Shaha, 1999). In contrast, Bagheri et al. found low levels of intelligence in strabismic patients with esotropia (Bagheri et al., 2013).

The present research had some restrictions like similar studies. The current study could not evaluated diplopia in children. Adult patients with diplopia often complain about difficulties to concentrate and to orientation. They have to close one eye to see things better, but this behavior can result in feelings of eye strain (Hatt, Leske, Bradley, Cole, & Holmes, 2009).

This may explain why diplopic adult patients also report psychosocial concerns since double vision may make them feeling tired, stressed and worried. It may be more important factor to affect the test passing. Moss TP has reviewed the literature relating to the visibility and severity of a disfigurement and psychological distress in adult. He concluded that there is no clear relation between the objective severity of a ‘‘visible difference’’ and adjustment (Moss, 2005).

In regards to strabisimic patients, ophthalmologist should observe not only the evidence of difficulties with daily visual functions but also related psychosocial concerns. We believe that improving attitude of society in relation to these children remains the most practical approach to decreasing psychological distress in future. With respect to the results obtained better support of parents and other care providers of children, particularly at home and, play an important role in decreasing the rate of the adulthood behavior disorder and intelligence deficiency.

## 5. Conclusion

In the present research, members of study and control groups had same socio-economic status. Therefore, it seems possible to face random homogeneity in respondents (which is one of the enhancing validity factors of statistical conclusion of studies). However, this study failed to show the effect of ocular misalignment on IQ and suggested that it is not a sufficient stimulus for eliciting IQ and any other factors such as tribes and paternal educational level may be more important factors.

This study demonstrated the importance of paternal educational level and tribe, regardless of ocular misalignment, because it cannot interfere with the functional well-being of the children. The complexity of the relationships between IQ, and strabismus warrant additional studies to clarify any cause-effect relationship.

In order to establish the longer term impact of ocular deviation and ocular surgery follow up questionnaires and interviews 2 years later are planned.
